# Contextual, maternal, and infant factors in preventable infant deaths: a statewide ecological and cross-sectional study in Rio Grande do SUL, Brazil

**DOI:** 10.1186/s12889-022-14913-z

**Published:** 2023-01-12

**Authors:** Ivete Maria Kreutz, Iná S. Santos

**Affiliations:** 1 Pontifícia Universidade Católica do Rio Grande do Sul (PUCRS), Escola de Medicina, Programa Pós-graduação Pediatria e Saúde da Criança, Porto Alegre, RS Brazil; 2 Universidade Federal de Pelotas, Programa de Pós-graduação em Epidemiologia, Pelotas, RS Brazil

**Keywords:** Infant mortality, Infant mortality rate, Preventable deaths

## Abstract

**Background:**

Ending preventable deaths of newborns and children under five by 2030 is among the United Nations Sustainable Development Goals. This study aimed to describe infant mortality rate due to preventable causes in Rio Grande do Sul (RS), the Southernmost state in Brazil. With 11,329,605 inhabitants and 141,568 live births in 2017, RS was the fifth most populous state in the country.

**Method:**

An ecological and cross-sectional statewide study, with data extracted from records of the Mortality Information System, Death Certificates, and Live Birth Certificates for the year 2017. Preventability was estimated by applying the List of Causes of Deaths Preventable through Intervention of SUS (acronym for Sistema Unico de Saude - Brazilian Unified Health System) Intervention. Rates of preventable infant mortality (PIMR), preventable early neonatal mortality (PENMR), preventable late neonatal mortality (PLNMR), and preventable post-neonatal mortality (PPNMR) per 1000 live births (LB) were quantified. Incidence ratios, according to contextual characteristics (human development index of the health region and of the municipality; Gini index of the municipality), maternal characteristics at the time of delivery (age, education, self-reported skin color, presence of a partner, number of antenatal care consultations, and type of delivery), and characteristics of the child at the time of birth (gestational age, weight, and pregnancy type) were calculated.

**Results:**

In 2017, there were 141,568 live births and 1425 deaths of infants younger than 1 year old, of which 1119 were preventable (PIMR = 7.9:1000 LB). The PENMR, PLNMR, and PPNMR were 4.1:1000 LB; 1.5:1000 LB; and 2.3:1000 LB, respectively. More than 60% of deaths in the first week and 57.5% in the late neonatal period could be reduced through adequate care of the woman during pregnancy. The most frequent preventable neonatal causes were related to prematurity, mainly acute respiratory syndrome, and non-specified bacterial septicemia. In the post-neonatal period, 31.8% of deaths could be prevented through adequate diagnostic and treatment.

**Conclusions:**

The strategies needed to reduce preventable infant deaths should preferably focus on preventing prematurity, through adequate care of the woman during pregnancy.

## Introduction

Globally, the estimated infant mortality rate (IMR) decreased 55.4% between 1990 and 2018, falling from 65:1000 live births (LB) to 29:1000 LB [1]. In Brazil, between 1982 and 2015, IMR decreased more than 80% (from 71.3:1000 LB to 14.0:1000 LB), placing the country among those that successfully met objective number four of the Millennium Development Goals, whose target was a two-thirds reduction in the mortality of infants under 5 years old, between 1990 and 2015 [2, 3]. The sharp decline in infant mortality in Brazil in the period was due to the expansion of access to prenatal care and hospital care for childbirth in the SUS (acronym in Portuguese for Unified Health System - Sistema Unico de Saúde), promotion of breastfeeding, increased vaccination coverage, monitoring of the child’s growth and development in the first year of life, and reduction in geographical and economic inequalities in the country, which still persist nonetheless [4–6]. In 2017, the IMR in Brazil was 13.4:1000 LB, with the South (where the Rio Grande do Sul state is located) and Southeast regions of the country showing rates below the national average (10.1 and 11.3:1000 LB, respectively), while in the North (15.4:1000 LB), Northeast (14.1:1000 LB), and Central-West region (11.7:1000 LB) the means were higher [7]. The mortality rate declined from 2017 to 2020, and this variation was higher in the early neonatal period (0–6 days after birth) [8]. In 2020, the mortality rates were 6.5, 2.1, 3.4, and 1.7 per 1000 LB, respectively, for the early neonatal, late neonatal (7–27 days), post-neonatal (28–364 days), and 1–4 years of age periods [8].

Among the United Nations Sustainable Development Goals, of which Brazil is a signatory, there is the goal of ending preventable deaths of newborns and children under five by 2030 [1]. Achieving that goal involves monitoring infant mortality, with the aim of accompanying the changes in mortality rates over time, evaluating the circumstances that led to deaths, as well as proposing measures to improve the quality of healthcare and other actions to reduce infant mortality [9]. When approached under a territorial reference, infant mortality rate has allowed to put in evidence existing social inequalities, enabling better targeting of public health interventions [10]. Infant mortality according to preventable causes, in this context, is considered a “sentinel event” of the quality of health care, being important to identify the spatial variations and territorial inequalities of this indicator. Thus, this study aimed to describe IMR and its components (early neonatal mortality, late neonatal mortality, and post-neonatal mortality) due to preventable causes in Rio Grande do Sul, in 2017, according to contextual, maternal, and infant characteristics. Our hypothesis was that preventable causes accounted for most of the infant deaths and were related to contextual, maternal and child characteristics at birth.

## Methods

This was a descriptive ecological and cross-sectional statewide study conducted in Rio Grande do Sul (RS), the Southernmost state in Brazil. With 11,329,605 inhabitants living in 497 municipalities in 2017, distributed in 30 Health Regions, RS was the fifth most populous state in the country [11].

Infant deaths at RS in 2017 were identified at the Mortality Information System (SIM, acronym in Portuguese for Sistema de Informação sobre Mortalidade) [12]. The SIM, developed by the Ministry of Health in 1975, is the product of the unification of more than forty Death Certificate (DC) models used over the years to collect data on mortality in the country. With its long time series, the SIM is a national asset, containing fundamental information on causes of illness that led to death. It is also one of the main instruments to support the development of more effective public health and social security policies aimed at prevention, promotion, and health care. The records from the SIM contain socioeconomic data, place of residence and occurrence, fetal and non-fetal deaths, conditions and causes of death, and information on external causes.

Preventable deaths were defined according to the List of Causes of Deaths Preventable through Intervention of the SUS, which classifies the deaths of infants under five into three categories: preventable causes, ill-defined causes, and other causes (not clearly preventable) [13]. Preventable causes are classified according to six subgroups: through immunoprevention actions; through adequate care of the woman during pregnancy; through adequate care of the woman during delivery; through adequate care of the newborn; through adequate diagnostic and treatment actions; and through adequate health promotion actions, linked to adequate healthcare actions.

To classify the cause of death, information extracted from Death Certificates (DC) and Live Birth Certificates (LBC) was used. From the DC, the disease or morbid state that directly caused the death, the antecedent causes (the morbid states, if any, that produced the direct cause of death) and the underlying cause of death were extracted. From the LBC, maternal and child variables at birth were extracted. For classification as to preventability, the DC data were analyzed in the light of the LBC information. Thus, for example, a child born at 30 weeks of gestational age, whose DC had the respiratory distress syndrome of the newborn as the underlying cause of death, this death was attributed to premature birth (preventable by attention to the woman during pregnancy).

In each case, the utilization of prenatal care by the pregnant woman (absence of prenatal care, late-onset and/or incomplete prenatal care) and access to primary health care, specialized outpatient care, specialized hospital care, high-risk maternity hospital and neonatal and/or pediatric intensive care unit at the place of residence were considered. The classification of causes of death was conducted separately by the two authors and the disagreements discussed until the consensus.

### Independent variables

The selection of the independent variables took into account the distal (social), intermediate (care) and proximal (biological) risk factors for infant mortality, based in the model proposed by Mosley and Chen [14].

### Contextual characteristics

The Human Development Index (HDI) of the Health Region, the HDI and the Gini index of the municipality of residence of the family were investigated. The HDI evaluates the quality of life and economic development of a population, and varies between 0 (no human development) to 1 (total human development) [15]. HDI is classified as very high (0.800–1.0), high (0.700–0.799), medium (0.600–0.699), low (0.500–0.599), and very low (0.000–0.499). The Health Regions of Rio Grande do Sul are classified into only two categories: high and medium. Among the municipalities, only one has a very high HDI (the state capital, Porto Alegre), one has a low HDI (Dom Feliciano), and none are in the very low category. For the analyses, the HDI of the municipalities was categorized as “very high/high” and “medium/low.”

The Gini index measures the income concentration in a particular group, indicating the difference between the incomes of the poorest and that of the richest. It varies from 0 (situation of equality) to 1 (one person holds all the wealth) [15]. For the analyses, the Gini index was categorized into quartiles, where the 1st quartile was represented by the lowest values (0.2841–0.4333) and the 4th quartile accounted for the highest ones (0.5194–0.7248).

### Maternal and infant characteristics

Maternal and infant variables at the time of birth were extracted from the Live Birth Certificates (LBC). The information on the mothers included: age in full years at the time of delivery; full years of schooling (subsequently categorized as 0–7, 8–11, and ≥ 12); marital status, reported by the mother as single, married, widow, legally separated/divorced, stable union, or ignored (later recoded as “with a partner,” which included married women and those in a stable union, and “without a partner,” corresponding to the remaining categories); self-reported skin color (white, black, yellow, brown, and indigenous) – due to the small number of women who declared themselves as yellow (*N* = 137) or indigenous (*N* = 726), the colors yellow, brown, and indigenous were grouped in the same category (“mixed”); number of antenatal consultations, categorized as 0, 1–3, 4–6, and ≥ 7); and type of birth (vaginal or caesarean).

The information on the infants included sex (male or female); gestational age – for the analyses categorized as < 28, 28–31, 32–36, and ≥ 37 weeks of gestation; low birth weight (LBW; < 2500 g) (yes or no); and type of pregnancy (single or multiple).

### Analysis

The information extracted from the official documents were entered onto an Excel spreadsheet, specifically built for the study, and subsequently analyzed in the Stata 12.1 program (Stata Corp., College Station, USA) [16]. The IMR, early neonatal mortality rate (ENMR), late neonatal mortality rate (LNMR), and post-neonatal mortality rate (PNMR) were calculated by dividing, respectively, the number of deaths occurring between 0 and 364, 0–6, 7–27, and 28–364 days of life by the number of LB in 2017, and multiplying by 1000.

Afterwards, the IMR, ENMR, LNMR, and PNMR due to preventable deaths (respectively PIMR, PENMR, PLNMR, and PPNMR) were calculated, whose numerators were the number of preventable deaths occurring in the respective age groups. The number and the proportion of preventable deaths, according to the preventability classification were obtained for each age group. The most frequent causes of preventable deaths were recorded. The incidence, differences in incidence, and the gross cumulative incidence ratio according to the independent variables were calculated. Chi-square test or 95% confidence interval (95% CI) was used to assess the statistical significance of the observed differences in infant mortality rates between categories of the independent variables.

## Results

At RS, in 2017, there were 141,568 live births and 1425 deaths of infants under 1 year old, corresponding to an IMR of 10.1:1000 LB (Table 1). Half of the deaths occurred in the first week of life, corresponding to an ENMR of 5.0:1000. The LNMR and PNMR were 1.9:10000 LB and 3.1:1000 LB, respectively. Regarding preventability, it was possible to classify the cause of 1421 of the deaths, 1119 of which were found to be preventable, corresponding to a PIMR of 7.9:1000 LB. The PENMR, PLNMR, and PPNMR were 4.1:1000 LB; 1.5:1000 LB; and 2.3:1000 LB, respectively.

**Table 1:** Infant mortality and preventable infant mortality rate per 1000 live births with 95% confidence interval (95% CI), according to contextual, maternal, and infant characteristics. Rio Grande do Sul state, 2017.

Variables	Number of live births	IMR (95% CI)	PIMR (95% CI)	ENMR (95% CI)	PENMR (95% CI)	LNMR (95% CI)	PLNMR (95% CI)	PNMR (95% CI)	PPNMR (95% CI)
Total	141,568	10.1 (9.5–10.6)	7.9 (7.4–8.4)	5.0 (4.7–5.4)	4.1 (3.8–4.5)	1.9 (1.7–2.2)	1.5 (1.3–1.7)	3.1 (2.8–3.4)	2.3 (2.0–2.5)
*HDI of the health region (N = 30)*									
High	103,773	9.7 (9.1–10.3)	7.6 (7.1–8.2)	4.8 (4.4–5.2)	3.9 (3.5–4.3)	1.9 (1.6–2.1)	1.4 (1.2–1.7)	3.1 (2.7–3.4)	2.3 (2.0–2.6)
Medium	37,795	11.0 (10.0–12.1)	8.6 (7.7–9.6)	5.7 (5.0–6.5)	4.8 (4.2–5.6)	2.2 (1.7–2.7)	1.5 (1.2–2.0)	3.1 (2.7–3.8)	2.2 (1.8–2.8)
*Characteristics of the municipality*									
HDI (*N* = 497)									
Very High/High	122,183	10.0 (9.5–10.6)	7.9 (7.4–8.4)	5.0 (4.6–5.4)	4.1 (3.8–4.5)	2.0 (1.7–2.2)	1.5 (1.3–1.7)	3.1 (2.8–3.4)	2.3 (2.0–2.6)
Medium/Low	19,316	10.0 (8.7–11.5)	7.8 (6.6–9.1)	5.2 (4.2–6.3)	4.2 (3.3–5.2)	1.7 (1.1–2.3)	1.2 (0.8–1.8)	3.2 (2.5–4.1)	2.3 (1.7–3.1)
Gini Index (quartiles) (*N* = 497)									
1st quartile (lowest)	11,893	9.4 (7.8–11.3)	7.5 (6.0–9.2)	5.0 (3.8–6.4)	4.0 (3.0–5.3)	1.6 (1.0–2.5)	1.3 (0.7–2.1)	2.9 (2.0–4.0)	2.3 (1.5–3.3)
2nd	29,619	10.3 (9.2–11.5)	7.7 (6.7–8.8)	5.3 (4.5–6.2)	4.1 (3.4–4.9)	2.0 (1.5–2.6)	1.4 (1.0–1.9)	3.0 (2.4–3.7)	2.2 (1.7–2.8)
3rd	36,977	9.6 (8.6–10.6)	7.4 (6.6–8.3)	5.1 (4.4–5.9)	4.1 (3.5–4.8)	1.6 (1.2–2.0)	1.3 (1.0–1.7)	2.9 (2.4–3.5)	2.0 (1.6–2.5)
4th quartile (highest)	63,032	10.3 (9.5–11.1)	8.4 (7.7–9.1)	4.9 (4.4–5.5)	4.2 (3.7–4.7)	2.2 (1.8–2.6)	1.7 (1.4–2.0)	3.2 (2.8–3.7)	2.5 (2.1–2.9)
*Maternal characteristics*									
Age (years) (*N* = 141,566)									
< 20	18,163	12.0 (10.5–13.7)	10.1 (8.7–11.6)	6.1 (5.0–7.3)	5.3 (4.3–6.4)	2.0 (1.4–2.7)	1.6 (1.1–2.3)	3.9 (3.0–4.9)	3.2 (2.4–4.1)
20–29	65,589	9.9 (9.1–10.7)	7.9 (7.2–8.6)	5.1 (4.6–5.7)	4.1 (3.6–4.6)	1.9 (1.6–2.3)	1.4 (1.1–1.7)	3.0 (2.6–3.4)	2.3 (1.9–2.7)
30–34	32,124	8.6 (7.6–9.7)	6.6 (5.7–7.5)	4.4 (3.7–5.2)	3.5 (2.9–4.2)	1.8 (1.4–2.3)	1.5 (1.1–2.0)	2.4 (1.9–3.0)	1.6 (1.2–2.1)
≥ 35	25,690	9.4 (8.2–10.6)	6.7 (5.7–7.8)	4.3 (3.5–5.1)	3.4 (2.7–4.2)	2.0 (1.5–2.6)	1.3 (0.9–1.8)	3.1 (2.5–3.9)	2.0 (1.5–2.6)
Schooling (years) (*N* = 141,211)									
0–7	23,794	14.0 (12.5–15.6)	10.8 (9.5–12.2)	6.3 (5.3–7.4)	5.0 (4.1–6.0)	2.6 (2.0–3.3)	1.9 (1.4–2.5)	5.1 (4.2–6.1)	3.9 (3.2–4.8)
8–11	82,760	9.5 (8.8–10.2)	7.5 (6.9–8.1)	5.0 (4.5–5.5)	4.1 (3.7–4.5)	1.8 (1.5–2.1)	1.3 (1.1–1.6)	2.8 (2.4–3.2)	2.1 (1.8–2.4)
≥ 12	34,657	7.1 (6.2–8.0)	5.5 (4.8–6.3)	3.6 (3.0–4.3)	3.0 (2.4–3.6)	1.6 (1.2–2.1)	1.3 (0.9–1.7)	2.0 (1.5–2.5)	1.4 (1.0–1.9)
Living with a partner (*N* = 141,109)									
Yes	67,789	8.7 (8.0–9.4)	6.5 (5.9–7.1)	4.2 (3.7–4.7)	3.3 (2.9–3.8)	2.0 (1.7–2.4)	1.5 (1.2–1.8)	2.5 (2.1–2.9)	1.7 (1.4–2.0)
No	73,320	10.4 (9.7–11.2)	8.4 (7.7–9.1)	5.3 (4.8–5.8)	4.5 (4.0–5.0)	1.8 (1.5–2.1)	1.3 (1.0–1.6)	3.3 (2.9–3.7)	2.5 (2.1–2.9)
Skin color (*N* = 141,097)									
White	117,223	10.0 (9.4–10.6)	7.8 (7.3–8.3)	5.0 (4.6–5.4)	4.1 (3.7–4.5)	1.9 (1.7–2.2)	1.5 (1.3–1.7)	3.1 (2.8–3.4)	2.3 (2.0–2.6)
Mixed	14,076	8.0 (6.6–9.6)	6.6 (5.3–8.1)	4.0 (3.0–5.2)	3.6 (2.7–4.8)	1.6 (1.0–2.4)	1.1 (0.6–1.8)	2.4 (1.7–3.4)	1.9 (1.3–2.8)
Black	9798	5.5 (4.1–7.2)	4.5 (3.3–6.0)	2.1 (1.3–3.3)	1.8 (1.1–2.9)	1.1 (0.6–2.0)	0.9 (0.4–1.7)	2.2 (1.4–3.4)	1.7 (1.0–2.8)
No. antenatal care consultations (*N* = 141,356)									
0	2563	32.8 (26.2–40.4)	29.7 (23.4–37.0)	24.2 (18.6–30.9)	22.6 (17.2–29.2)	3.9 (1.9–7.2)	3.5 (1.6–6.6)	4.7 (2.4–8.2)	3.5 (1.6–6.6)
1–3	6092	41.9 (37.0–47.0)	36.8 (32.2–41.8)	22.6 (19.1–26.7)	20.2 (16.8–24.0)	7.7 (5.7–10.2)	7.4 (5.4–9.9)	11.5 (9,0–14.5)	9.2 (6.9–11.9)
4–6	24,462	18.8 (17.1–20.6)	15.9 (14.4–17.5)	9.9 (8.7–11.2)	8.5 (7.4–9.7)	3.9 (3.1–4.7)	3.3 (2.6–4.1)	4.9 (4.1–5.9)	4.1 (3.3–5.0)
≥ 7	108,239	5.1 (4.7–5.5)	3.4 (3.1–3.8)	2.1 (1.8–2.4)	1.5 (1.3–1.7)	1.0 (0.8–1.2)	0.6 (0.5–0.8)	1.9 (1.6–2.2)	1.3 (1.1–1.5)
Type of delivery (*N* = 141,530)									
Vaginal	52,551	11.0 (10.1–11.9)	9.4 (8.8–10.0)	6.2 (5.7–6.7)	5.6 (5.2–6.1)	1.7 (1.5–2.0)	1.4 (1.2–1.6)	3.1 (2.8–3.4)	2.5 (2.2–2.8)
Caesarean	88,979	8.9 (8.3–9.5)	6.5 (6.0–7.0)	4.1 (3.7–4.5)	3.1 (2.7–3.5)	2.0 (1.7–2.3)	1.5 (1.2–1.8)	2.9 (2.6–3.3)	2.0 (1.7–2.3)
*Characteristics of the infant at birth*									
Sex (N = 141,541)									
Male	72,946	11.1 (10.4–11.9)	8.7 (8.0–9.4)	5.4 (4.9–6.0)	4.3 (3.8–4.8)	2.3 (2.0–2.7)	1.8 (1.5–2.1)	3.4 (3.0–3.8)	2.6 (2.2–3.0)
Female	68,615	8.9 (8.2–9.6)	7.1 (6.5–7.7)	4.6 (4.1–5.1)	3.9 (3.4–4.4)	1.6 (1.3–1.9)	1.2 (0.9–1.5)	2.8 (2.4–3.2)	2.0 (1.7–2.4)
Gestational age (weeks) (*N* = 140,980)									
< 28	743	585.5 (549.1–621.2)	574.7 (538.2–610.6)	409.2 (373.5–445.5)	401.1 (365.6–437.3)	118.4 (96.1–143.9)	118.4 (96.1–143.9)	57.9 (42.2–77.2)	55.2 (39.9–74.1)
28–31	1435	138.7 (121.2–157.6)	115.0 (98.9–132.6)	83.0 (69.2–98.4)	63.4 (51.4–77.3)	30.7 (22.4–40.9)	29.3 (21.2–39-4)	25.1 (7.6–34.6)	22.3 (15.3–31.3)
32–36	14,421	17.5 (15.4–19.7)	10.5 (8.9–12.3)	8.4 (7.0–10.0)	4.8 (3.7–6.0)	3.3 (2.4–4.4)	2.1 (1.4–3.0)	5.8 (4.6–7.2)	3.5 (2.6–4.6)
≥ 37	124,381	3.9 (3.6–4.3)	2.7 (2.4–3.0)	1.2 (1.0–1.4)	0.9 (0.7–1.1)	0.7 (0.6–0.9)	0.3 (0.2–0.4)	2.0 (1.8–2.3)	1.5 (1.3–1.7)
Low birth weight (< 2500 g) (*N* = 141,558)									
Yes	13,279	72.0 (67.6–76.5)	59.1 (55.2–63.3)	43.1 (39.9–46.9)	36.1 (33.0–39.4)	14.3 (12.4–16.5)	12.4 (10.6–14.4)	14.6 (12.6–16.8)	10.6 (8.9–12.5)
No	128,279	3.6 (3.3–3.9)	2.6 (2.3–2.9)	1.1 (0.9–1.3)	0.8 (0.6–1.0)	0.6 (0.5–0.7)	0.3 (0.2–0.4)	1.9 (1.7–2.2)	1.4 (1.2–1.6)
Type of pregnancy (*N* = 141,534)									
Single	138,067	9.0 (8.5–9.5)	6.9 (6.5–7.3)	4.3 (4.0–4.7)	3.5 (3.2–3.8)	1.8 (1.6–2.0)	1.3 (1.1–1.5)	2.9 (2.6–3.2)	2.1 (1.9–2.4)
Multiple	3467	41.5 (35.1–48.7)	38.1 (31.9–45.0)	28.5 (23.3–34.6)	27.1 (22.0–33.1)	6.9 (4.4–10.3)	6.3 (4.0–9.6)	6.1 (3.7–9.2)	4.6 (2.6–7.5)

*IMR* infant mortality rate, *ENMR* early neonatal mortality rate, *LNMR* late neonatal mortality rate, *PNMR* post-neonatal mortality rate, *PIMR* preventable infant mortality rate, *PENMR* preventable early neonatal mortality rate, *PLNMR* preventable late neonatal mortality rate, *PPNMR* preventable post-neonatal mortality rate

**Fig. 1:**
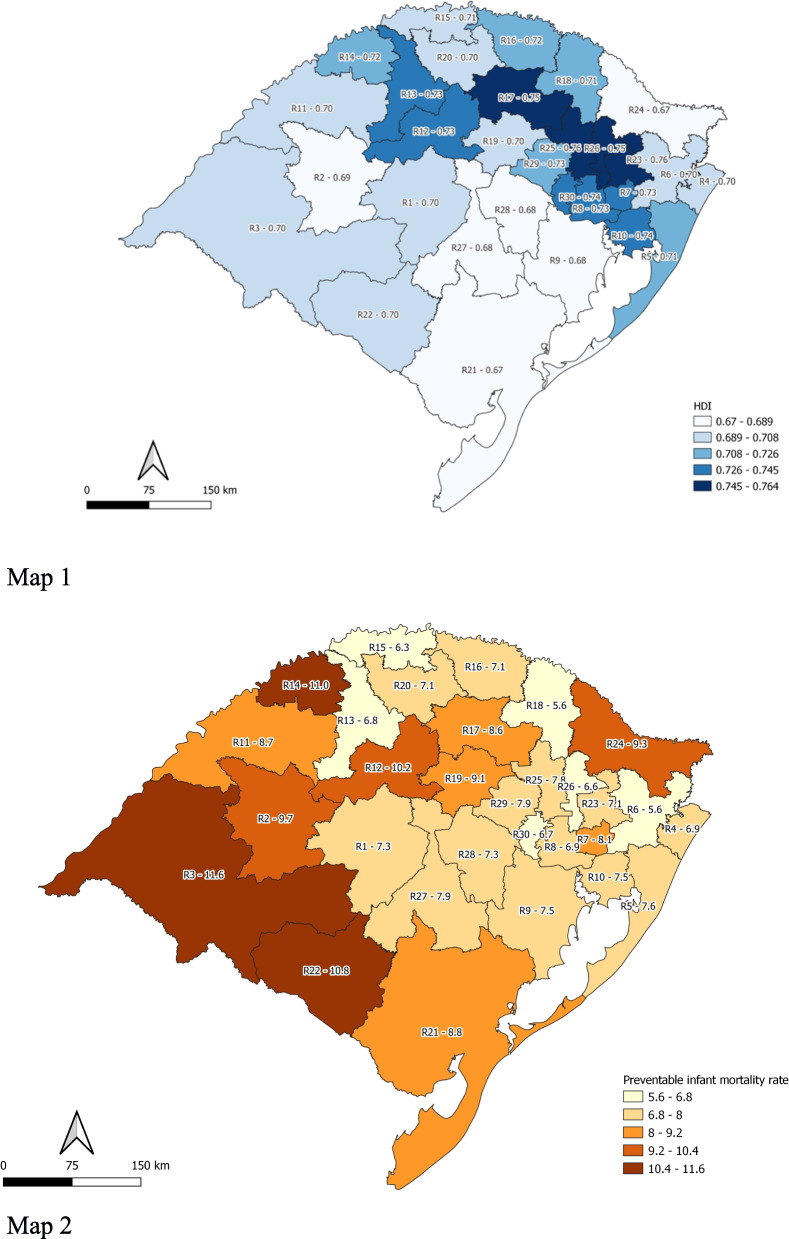
Map 1: HDI of the 30 health regions of Rio Grande do Sul; Map 2: Preventable infant mortality per 1000 live births, in every health region of Rio Grande do Sul, in 2017

Maps 1 and 2 (Fig. 1) show the mean HDI and the PIMR of each one of 30 Health Regions in the state. The PIMR varied from 5.7:1000 LB in the 6th Health Region to 11.6:1000 LB in the 3rd Health Region.

Table 1 presents the mortality rates and preventable mortality rates by age of death, according to contextual, maternal, and infant characteristics at birth. Missing information was below 0.5% for all variables. Variables with higher missing data were gestational age (0.41%), maternal skin color (0.33%), presence of a partner (0.32%), maternal schooling (0.25%), and number of antenatal consultations (0.15%).

There was no difference in preventable mortality rates at any age according to the HDI of the Health Region nor according to the HDI or the Gini Index of the municipality of residence of the family. The PIMR was higher among male than in female infants (PIMR = 8.7; 95% CI 8.0–9.4:1000 LB versus PIMR = 7.1; 95% CI 6.5–7.7:1000 LB, respectively); among infants from adolescent mothers (PIMR = 10.1; 95% CI 8.7–11.6:1000 LB), in comparison with those from mothers aged 20–29 (PIMR = 7.9; 95% CI 7.2–8.6:1000 LB), 30–34 (PIMR = 6.6; 95% CI 5.7–7.5:1000 LB) and 35 years or more (PIMR = 6.7; 95% CI 5.7–7.8:1000 LB); and with a lower educational level (PIMR = 10.8; 95% CI 9.5–12.2:1000 LB), in comparison with those of mothers with 8–11 years of schooling (PIMR = 7.5; 95% CI 6.9–8.1:1000 LB) and ≥ 12 years (PIMR = 5.5; 95% CI 4.8–6.3:1000 LB). There was no difference in PIMR between infants from white (PIMR = 7.8; 95% CI 7.3–8.3: 1000 LB) and mixed mothers (PIMR = 6.6; 95% CI 5.3–8.1:1000 LB) nor between infants from mixed and black mothers (PIMR = 4.5; 95% CI 3.3–6.0:1000 LB), whereas PIMR was higher among children of white mothers in comparison to those from black mothers, specifically at the early neonatal period (PENMR = 4.1; 95% CI 3.7–4.5:1000 LB in infants from white mothers and PENMR = 1.8; 95% CI 1.1–2.9:1000 LB in those from black mothers). The PIMR was higher among infants of mothers who lived without a partner (PIMR = 8.4; 95% CI 7.7–9.1:1000 LB) than among those who had a partner (PIMR = 6.5; 95% CI 5.9–7.1:1000 LB). Among children of mothers who did not attend or who attended 1–3 antenatal care consultations, the preventable mortality rate at all age groups was higher than among those who attended ≥7 consultations. The PIMR and PENMR were higher among the children born via vaginal delivery, in comparison with those born via caesarean (9.4 versus 6.5:1000 LB, and 5.6 versus 3.1: 1000 LB, respectively). Preterm and LBW infants, as well as those from multiple pregnancies presented the highest preventable mortality rates at all ages, especially in the early neonatal period.

Table 2 shows that more than three quarters of the deaths (*N* = 1119; 78.5%) were preventable with the resources available in the SUS. That proportion was even higher among the deaths in the first week of life, corresponding to 82.2% of all deaths in that age group (586/713). More than 60% of deaths in the first week (444/689) and 57.5% of those occurring in the late neonatal period (158/275) were reducible through adequate care of the woman during pregnancy. In the post-neonatal period, almost one third (31.8%) of the deaths (139/437) were preventable through adequate diagnostic and treatment actions.

**Table 2:** Preventability of the cause of infant death at Rio Grande do Sul state in 2017 (*N* = 1425).

	Age of the child at death (days)							
	0–6		7–27		28–364		Total	
	N	% (95% CI)	N	% (95% CI)	N	% (95% CI)	N	% 95 CI
*Reducible through*								
*Immunoprevention*	0	0.0% (0.0–0.0%)	0	0.0% (0.0–0.0%)	9	2.1% (0.9–3.9%)	9	0.6% (0.3–1.2%)
*Care of the woman during pregnancy*	444	62.3% (58.6–65.8%)	158	57.4% (51.4–63.4%)	87	19.9% (16.3–24.0%)	689	48.3% (45.7–50.9%)
*Care of the woman during delivery*	62	8.7% (6.7–11.0%)	6	2.2% (0.8–4.7%)	9	2.1% (0.9–3.9%)	77	5.4% (4.3–6.7%)
*Care of the newborn*	78	10.9% (8.7–13.5%)	41	14.9% (10.9–19.7%)	2	4.6% (2.8–7.0%)	121	8.5% (7.1–10.1%)
*Diagnosis and treatment*	0	0.0% (0.0–0.0%)	2	0.7% (0.09–2.6%)	139	31.8% (27.5–36.4%)	141	9.9% (8.4–11.6%)
*Health promotion*	2	0.3% (1.8–4.5%)	2	0.7% (0.09–2.6%)	78	17.8% (14.4–21.8%)	82	5.8% (4.7–7.2%)
Total preventable	586	82.2% (79.2–84.9%)	209	76.0% (70.5–80.9%)	324	74.1% (69.8–78.2%)	1119	78.5% (76.3–80.6%)
*Not clearly reducible*	125	17.5% (14.8–20.5%)	65	23.6% (18.7–29.1%)	112	25.6% (21.6–30.0%)	302	21.2% (19.1–23.4%)
*Ill-defined*	2	0.3% (0.0–1.0%)	1	0.4% (0.01–2.0%)	1	0.2% (0.01–1.3%)	4	0.3% (0.08–0.7%)
***Total deaths***	713		275		437		1425	

*95% CI* 95% confidence interval

The most frequent preventable neonatal causes of death were related to prematurity: acute respiratory syndrome in the newborn (*N* = 80), non-specified bacterial septicemia in the newborn (*N* = 73), fetus and newborn affected by hypertensive maternal disorders (*N* = 44), fetus and newborn affected by chorioamnionitis (*N* = 35), and fetus and newborn affected by premature rupture of the membranes (*N* = 34). Almost a quarter (24.7%; *N* = 352) of the 1425 deaths were of infants with some malformation or genetic syndrome. Malformations of the cardiovascular system were the most prevalent (44.6%; *N* = 157), and genetic syndromes were present in 9.6% (*N* = 34) of those deaths.

Table 3 describes the incidence, the absolute difference in percentage points (p.p.), and the cumulative incidence ratio (with 95% confidence interval - 95%CI) of the preventable deaths, according to the independent variables. The absolute difference in the incidence varied from 0.01 p.p. (among children of mothers aged 30–34 years, in comparison with those of mothers aged ≥35) to 57.20 p.p. (among those born at < 28 weeks of gestation, in comparison with those born at ≥37 weeks of gestation). The preventable deaths were 16% more frequent in Health Regions with a medium HDI than in Regions with a high HDI; and 44% less frequent in municipalities with a medium/low HDI, in comparison with those with a very high/high HDI. Preventable deaths were 3.11 times more frequent in municipalities from the 4th quartile and 7.50 times more frequent in municipalities from the 1st quartile of the Gini Index, in comparison with those at the 2nd quartile.

**Table 3:** Cumulative incidence of preventable infant deaths, according to contextual, maternal, and infant characteristics. Rio Grande do Sul state, 2017.

	Number of live births	Preventable deaths N (%)	Difference in incidence (p.p.)^1^	Gross cumulative incidence ratio (95% CI)^2^
*HDI of the health region*		*p* = 0,028		
High	94,903	793 (0.84)	Ref.	1.00
Medium	33,590	326 (0.97)	0.13	1.16 (1.02–1.32)
*Characteristics of the municipality*				
HDI		*p* < 0.001		
Very high/high	110,830	972 (0.88)	Ref.	1.00
Medium/low	30,709	151 (0.49)	0.39	0.56 (0.47–0.67)
Gini index (quartiles)		p < 0.001		
1st quartile	2750	89 (3.24)	2.81	7.50 (5.89–9.54)
2nd	53,504	231 (0.43)	Ref.	1.00
3rd	35,730	282 (0.79)	0.36	1.83 (1.54–2.17)
4th quartile	38,839	521 (1.34)	0.91	3.11 (2.66–3.63)
*Maternal characteristics*				
Age (years)		*p* = 0.005		
< 20	18,163	183 (1.01)	0.34	1.50 (1.22–1.85)
20–29	65,589	516 (0.08)	0.59	0.13 (0.99–1.40)
30–34	32,124	211 (0.66)	0.01	0.98 (0.80–1.20)
≥ 35	25,690	172 (0.67)	Ref.	1.00
Schooling (years)		*p* = 0.738		
0–7	23,794	258 (1.08)	0.53	1.98 (1.64–2.38)
8–11	82,760	623 (0.75)	0.20	1.37 (1.17–1.61)
≥ 12	34,657	190 (0.55)	Ref.	1.00
Living with a partner		*p* = 0.010		
Yes	67,789	441 (0.65)	Ref.	1.00
No	73,320	614 (0.84)	0.19	1.29 (1.14–1.45)
Skin color		*p* = 0.612		
White	117,223	918 (0.78)	Ref.	1.00
Mixed	14,076	93 (0.66)	0.12	0.84 (0.68–1.04)
Black	9798	44 (0.45)	0.33	0.57 (0.42–0.78)
No. antenatal care consultations		p < 0.001		
0	2563	76 (2.97)	2.63	8.84 (6.93–11.29)
1–3	6092	224 (3.68)	3.34	10.96 (9.30–12.92)
4–6	24,462	388 (1.59)	1.25	4.73 (4.10–5.45)
≥ 7	108,239	363 (0.34)	Ref.	1.00
Type of delivery		p < 0.001		
Vaginal	52,551	496 (0.94)	Ref.	1.00
Caesarean	88,979	580 (0.65)	0.29	0.69 (0.61–0.78)
*Characteristics of the infant at birth*				
Sex		*p* = 0.667		
Male	72,946	633 (0.87)	0.16	1.22 (1.09–1.38)
Female	68,615	486 (0.71)	Ref.	1.00
Gestational age (weeks)		p < 0.001		
< 28	743	427 (57.47)	57.20	213.38 (188.58–241.44)
28–31	1435	165 (11.50)	11.23	42.69 (35.70–51.06)
32–36	14,421	152 (1.05)	0.78	3.91 (3.23–4.74)
≥ 37	124,381	335 (0.27)	Ref.	1.00
Low birth weight (< 2500 g)		p < 0.001		
Yes	13,279	748 (5.63)	5.37	21.63 (19.04–24.58)
No	128,279	334 (0.26)	Ref.	1.00
Type of pregnancy		p < 0.001		
Single	138,067	951 (0.69)	Ref.	1.00
Multiple	3467	132 (3.81)	3.12	5.53 (4.74–6.87)

^1^*p.p*. percentage points

^2^*95% CI* 95% confidence interval; *p*-values are from chi-square test

The preventable deaths were 50% more frequent among children of adolescent mothers than among those of mother aged 35 or more; and 4.73 to 10.96 times more frequent among children of mothers who attended < 7 antenatal care consultations (Table 3). Among children of mothers who lived without a partner, the incidence of preventable deaths was around 30% higher than among those of mothers who lived with a partner. Children born via caesarean presented a 31% lower probability of dying due to preventable causes. The highest cumulative incidence ratios occurred among the preterm births (213.38; 42.69; and 3.91 times more frequent among those born at < 28, 28–31, and 32–36 weeks of gestation, respectively, than among those born at term), among LBW infants (21.63 times higher than among those born with ≥2500 g), and from multiple pregnancies (5.53 times higher than among those from single pregnancies).

## Discussion

The main finding of this study is that more than three quarters of infant deaths occurring at RS in 2017 were preventable using the resources available in the SUS, most of them (61.6%) through adequate care of the woman during pregnancy to reduce the number of preterm births. Avoidable causes accounted for 82.2% of early neonatal deaths, 76.0% of late neonatal deaths, and 74.1% of post-neonatal deaths in 2017. Nonetheless, there was a different structure of causes of death when comparing the neonatal and the post-neonatal periods. Most deaths occurring in the neonatal period were preventable through adequate attention to women during pregnancy, whereas at the post-neonatal period the deaths were mainly preventable through diagnosis and treatment. Consistent with our findings, another study assessing preventable infant deaths in RS from 2000 through 2004 showed that, despite the absolute reduction in the number of infant deaths in the period, the proportion of preventable deaths remained above 60% [17].

Between 2003 and 2017, infant mortality rate in RS fell 63.5%, decreasing from 15.9:1000 LB to 10.1:1000 LB [18, 19]. This reduction, however, could even be greater since, as identified in our study, most of deaths were preventable. Although this persistence can be partly attributed to the improvement in the quality of the data, evaluated by the reduction in the percentage of ill-defined causes [20], and to the 13% increase in the national coverage of the SIM for deaths of those aged less than 1 year verified in the period (from 79.4 to 89.6%) [21], other factors need to be considered.

The characteristic most strongly related with infant death in our study was gestational age. The greatest proportion of preventable deaths occurring in the early neonatal period was due to complications of prematurity or other causes for which prematurity is a risk factor, particularly infections [22]. In 2017, 11.8% of the children born alive in RS were less than 37 weeks of gestation, most of which (86.9%) were late preterm births (34–36 weeks of gestation), which, although apparently less vulnerable due to their weight and size at birth, have a higher risk of dying in the first year of life than those born at term [23]. Preventable deaths in the late preterm group in our study was four times higher than among infants born at term.

Prevention of preterm births however is a challenging task, because preterm birth is a complex outcome, with a multifactor etiology, differing according to the gestational age at which it occurs, race, and characteristics of the population [24]. Besides spontaneous cases, preterm births can be due to medical indication secondary to maternal diseases or fetal suffering. Moreover, the over-medicalization of births and the sharp increase in caesareans have been blamed for the current epidemic of preterm births in Brazil [4, 5]. A systematic review showed rising trends in the prevalence of prematurity in the country since 1990 [25]. A similar trend of increasing prematurity is observed in RS. In 2019, fifteen Health Regions presented rates above the state average (12.15%) [26]. Between 1982 and 2015, preterm births increased sharply in RS, from 5.8 to 13.8% [27]. Data from the Birth Cohorts of Pelotas, a city located in the south of RS, showed that in the same period caesareans increased from 27.7 to 65.1%, and were responsible for 86.2% of all births in the wealthiest families, while among the poorest (who generally accumulate more factors for high risk pregnancies), the prevalence was 50.5% [28].

Besides gestational age, other characteristics of the infant at birth were strongly associated with preventable mortality: LBW and multiple pregnancies. LBW was the second factor most strongly related to avoidable deaths in our study. Consistent with data on gestational age, the prevalence of LWB has increased in RS. In 2019, the prevalence of LBW in fifteen of the Health Regions was above the state average (9.6%) [26]. Together with gestational age, LWB is one of the main determinants of morbidity and mortality in the first year of life, primarily during the neonatal period [29].

Infants from multiple pregnancies had a cumulative incidence of preventable deaths throughout the first year of life more than five times higher than that observed among infants from single pregnancies. Between 2007 and 2017, double and triple pregnancies in RS corresponded to 10 and 0.8%, respectively, of all pregnancies [7]. The perinatal mortality rate is two to three times higher in twins than in single newborns, primarily due to prematurity, restricted fetal growth, LWB, and intrapartum anoxia [30, 31]. Among the maternal characteristics investigated, the number of antenatal care consultation was the most strongly associated with preventable infant deaths. The relationship between preventable infant mortality and antenatal care, verified in this and in other studies, may relate both to the insufficient number of consultations and to the low quality of care received [31, 32]. Both factors contribute to death in the first days of the child’s life, commonly related to preventable causes [33, 34]. Nonetheless, the number of antenatal consultations depends on the duration of the pregnancy, opening up the possibility of reverse causality bias. Mothers whose pregnancies end prematurely (an outcome that is related to high infant mortality) [35] have fewer opportunities for consultations during the antenatal period than those whose pregnancies reaches 37 weeks or more.

PIMR and specifically PENMR were higher in infants from white mothers than in infants from black mothers. The white population is the majority in Rio Grande do Sul State [11], and white women have better education level and less pregnant adolescents than black mothers, two factors related to lower rates of infant mortality [36]. A nationwide population-based, retrospective cohort study found substantial ethnoracial inequalities in child mortality in Brazil, especially among the Indigenous and Black populations [37]. Thus, our finding reflects more the higher number of births to women that declared white skin color, as well as the lack of adjustment for confounders, than the risk of dying according to maternal skin color. Nonetheless, as the objective of our study was not to explore risk factors for preventable infant mortality but instead to describe the distribution of the infant mortality in the State, this finding reflects the real incidence of the deaths in Rio Grande do Sul and must be interpreted in light of this objective.

The higher incidence of preventable deaths among infants born to mothers with fewer years of schooling and to mothers who lived without a partner is consistent with findings from a population-based birth cohort carried-out in the south of Rio Grande do Sul, in which infant deaths were three times higher among infants from mothers with fewer years of formal education and six times higher among those born to women who lived without a partner [38]. In the same way, our finding of increased preventable infant mortality among infants from adolescent mothers is in agreement with results from a nationally representative data from India, which showed that the odds ratio of childhood mortality was comparatively higher among lower aged women (< 20 years old) [39].

Of the contextual characteristics of place of residence, the Gini index of the municipality presented the highest incidence ratios. Avoidable mortality ratio was higher in the extremes of the Gini index, mainly in municipalities with lower income concentration. This possibly reflects the situation of poor municipalities with lack of access to qualified maternal and child health assistance.

This study has strengths and limitations. Among the strengths is the use of official data from SIM, a system organized with the specific aim of monitoring vital statistics of the Brazilian population. The findings of our study can contribute to the planning of maternal and child health services to quality the assistance provided to the child and maternal group of citizens in RS and in other settings with similar characteristics. The use of List of Causes of Deaths Preventable through SUS Interventions [13], which aims to systematize the contribution of different factors to infant mortality, as well as evaluating the effectiveness of health services is another strength of the study [32]. The investigation of a wide range of variables, potentially associated with preventable deaths, is another positive aspect of the study. The main limitation was the unavailability of detailed information on the factors and circumstances that led to death. Also, as the objective of the study was to describe the incidence of infant mortality in Rio Grande do Sul, instead of exploring risk factors for infant mortality in the State, no adjustment for confounding factors was done. Thus, the incidence differences and ratios can be under or overestimated.

## Conclusion

Although RS has one of the lowest IMR in Brazil, avoidable deaths are still the main cause of infant mortality, especially those related to attention to women during pregnancy. Policies and programs directed at women’s reproductive health implemented in Brazil since 1984 have managed to achieve wide coverage, primarily among women who need it most [5], but there is still a lot to improve in terms of family planning and quality of antenatal care, in order to achieve the goal of ending preventable infant deaths by 2030.

## Data Availability

The dataset analyzed during the current study are not publicly available because it was based on confidential documental review of the Brazilian Mortality Information System. To have access to analyzed dataset it is required to have authorization from the Director of the Department of Information Technology Management of Secretariat of Health of Rio Grande do Sul.

## References

[1] Infant mortality. WHO. https://www.who.int/data/gho/data/themes/topics/indicator-groups/indicator-group-details/GHO/infant-mortality (2021). Accessed 23 Jun 2022.

[2] Lima JC, Mingarelli AM, Segri NJ, Zavala AAZ, Takano OA (2017). Estudo de base populacional sobre mortalidade infantil. Ciên Saúde Colet.

[3] França EB, Lansky S, Rego MAS, Malta DC, França JS, Teixeira R (2017). Principais causas da mortalidade na infância no Brasil, em 1990 e 2015: estimativas do estudo de Carga Global de Doença. Rev Bras Epidemiol.

[4] Victora CG, Aquino EM, do Carmo Leal M, Monteiro CA, Barros FC, Szwarcwald CL (2011). Maternal and child health in Brazil: progress and challenges. Lancet.

[5] Leal MC, Szwarcwald CL, Almeida PVB, Aquino EML, Barreto ML, Barros F (2018). Reproductive, maternal, neonatal and child health in the 30 years since the creation of the Unified Health System (SUS). Cien Saude Colet.

[6] Ichihara MY, Ferreira AJ, Teixeira CS, Alves FJO, Rocha AS, Diógenes VHD (2022). Mortality inequalities measured by socioeconomic indicators in Brazil: a scoping review. Rev Saude Publica.

[7] Informações em Saúde (TABNET). http://tabnet.datasus.gov.br/cgi/menu_tabnet_php.htm. Accessed 23 Jun 2021.

[8] Moura EC, Cortez-Escalante J, Lima RT, Cavalcante FV, Alves LC, Santos LM (2022). Mortality in children under five years old in Brazil: evolution from 2017 to 2020 and the influence of COVID-19 in 2020. J Pediatr.

[9] The State of the World’s Children | UNICEF. https://www.unicef.org/reports/state-of-worlds-children. Accessed 23 Jun 2022.

[10] Faria RM (2022). A mortalidade infantil no brasil do século XXI: dilemas do desenvolvimento territorial e as desigualdades regionais em saúde. Raega Espaço Geográfico em Análise.

[11] IBGE. População. https://www.ibge.gov.br/estatisticas/sociais/populacao.html. Accessed 23 Jun 2022.

[12] DATASUS - SIM - Sistema de Informação sobre Mortalidade. http://sim.saude.gov.br/default.asp. Accessed 23 Jun 2022.

[13] Malta DC, Duarte EC, MFd A, MAdS D, OLd MN, Ld M (2007). Lista de causas de mortes evitáveis por intervenções do Sistema Único de Saúde do Brasil.

[14] Mosley WH, Chen LC (1984). An analytical framework for the study of child survival in developing countries. Popul Dev Rev.

[15] Atlas Brasil. http://www.atlasbrasil.org.br/. Accessed 23 Jun 2022..

[16] STATACORP L.P (2011). Stata statistical software: release 12.0. College Station TSL. Stata statistical software: release 12.0.

[17] Óbitos infantis evitáveis no Rio Grande do Sul: diferenças entre os períodos neonatal e pós-neonatal | Jung | Indicadores Econômicos FEE http://200.198.145.164/index.php/indicadores/article/view/4067. Accessed 01 Jul 2022.

[18] Rio Grande do Sul registra a menor taxa de mortalidade infantil da história - Secretaria da Saúde. https://saude.rs.gov.br/rio-grande-do-sul-registra-a-menor-taxa-de-mortalidade-infantil-da-historia. Accessed 23 Jun 2022.

[19] Redução da mortalidade infantil avança no Rio Grande do Sul - Portal do Estado do Rio Grande do Sul. https://estado.rs.gov.br/reducao-da-mortalidade-infantil-avanca-no-rs. Accessed 23 Jun 2022.

[20] Brasil. Manual para investigação do óbito com causa mal definida. Ministério da Saúde. Secretaria da Vigilância em Saúde. Departamento de Análise de Situação em Saúde. Brasília; 2009. p. 48..

[21] Brasil. Indicadores de mortalidade que utilizam a metodologia do Busca Ativa - Indicadores, Ações e Programas - Acesso à Informação - DASNT - SVS/MS. Ministério da Saúde. 2019. http://svs.aids.gov.br/dantps/acesso-a-informacao/acoes-e-programas/busca-ativa/indicadores-de-saude/mortalidade/. Accessed 23 Jun 2022.

[22] Lawn JE, Gravett MG, Nunes TM, Rubens CE, Stanton C (2010). Global report on preterm birth and stillbirth (1 of 7): definitions, description of the burden and opportunities to improve data. BMC Pregnancy Childbirth.

[23] Santos IS, Matijasevich A, Silveira MF, Sclowitz IK, Barros AJ, Victora CG (2008). Associated factors and consequences of late preterm births: results from the 2004 Pelotas birth cohort. Paediatr Perinat Epidemiol.

[24] Gravett MG, Rubens CE, Nunes TM (2010). Global report on preterm birth and stillbirth (2 of 7): discovery science. BMC Pregnancy Childbirth.

[25] Silveira MF, Santos IS, Barros AJ, Matijasevich A, Barros FC, Victora CG (2008). Increase in preterm births in Brazil: review of population-based studies. Rev Saude Publica.

[26] Brasil. Plano Estadual de Saúde: 2020/2023. Grupo de Trabalho de Planejamento, Monitoramento e Avaliação da Gestão (Org.). Porto Alegre/RS; 2020. p. 522.

[27] Silveira MF, Victora CG, Horta BL, da Silva BG, Matijasevich A, Barros FC (2019). Low birthweight and preterm birth: trends and inequalities in four population-based birth cohorts in Pelotas, Brazil, 1982–2015. Int J Epidemiol.

[28] Barros AJ, Victora CG, Horta BL, Wehrmeister FC, Bassani D, Silveira MF (2019). Antenatal care and caesarean sections: trends and inequalities in four population-based birth cohorts in Pelotas, Brazil, 1982–2015. Int J Epidemiol.

[29] Luke B, Williams C, Minogue J, Keith L (1993). The changing pattern of infant mortality in the US: the role of prenatal factors and their obstetrical implications. Int J Gynecol Obstet.

[30] Buhling KJ, Henrich W, Starr E, Lubke M, Bertram S, Siebert G (2003). Risk for gestational diabetes and hypertension for women with twin pregnancy compared to singleton pregnancy. Arch Gynecol Obstet.

[31] Matijasevich A, Santos IS, Barros AJ, Menezes A, Albernaz EP, Barros FC (2008). Perinatal mortality in three population-based cohorts from Southern Brazil: trends and differences. Cad Saúde Pública.

[32] Ventura RN, Puccini RF, Silva NN, Silva EMK, Oliveira EM (2008). The expression of vulnerability through infant mortality in the municipality of Embu. Sao Paulo Med J.

[33] Maia LTS, Souza WV, Mendes ACG (2020). Individual and contextual determinants of infant mortality in Brazilian state capitals: a multilevel approach. Cad Saúde Pública.

[34] Atkinson TB (2020). Infant mortality: access and barriers to quality perinatal care in North Carolina. N C Med J.

[35] Sania A, Smith ER, Manji K, Duggan C, Masanja H, Kisenge R (2018). Neonatal and infant mortality risk associated with preterm and small for gestational age births in Tanzania: individual level pooled analysis using the intergrowth standard. J Pediatr.

[36] Vilanova CS, Hirakata VN, de Souza Buriol VC, Nunes M, Goldani MZ, da Silva CH (2019). The relationship between the different low birth weight strata of newborns with infant mortality and the influence of the main health determinants in the extreme south of Brazil. Popul Health Metrics.

[37] Rebouças P, Goes E, Pescarini J, Ramos D, Ichihara MY, Sena S (2022). Ethnoracial inequalities and child mortality in Brazil: a nationwide longitudinal study of 19 million newborn babies. Lancet Glob Health.

[38] Varela AR, Schneider BC, Bubach S, Silveira MF, Bertoldi AD, Duarte LSM (2019). Fetal, neonatal, and post-neonatal mortality in the 2015 Pelotas (Brazil) birth cohort and associated factors. Cad Saúde Pública.

[39] Mandal S, Chouhan P. How maternal age links to childhood mortality? A brief analysis from NFHS-4 (2015–2016), India. Vulnerable Child Youth Stud. 2022;17(4):368–75. 10.1080/17450128.2022.2058136.

[40] Brasil. Ministério da Saúde. Resolução No 466/12. Conselho Nacional de Saúde. 2012. https://conselho.saude.gov.br/resolucoes/2012/Reso466.pdf. Accessed 23 Jun 2022.

